# The impact of the COVID-19 pandemic on symptom subtypes of obsessive-compulsive disorder: a cross-sectional study

**DOI:** 10.1192/bjo.2021.679

**Published:** 2021-06-18

**Authors:** Athanasios Hassoulas, Katja Umla-Runge, Olivia Adams, Madeline Scurlock-Green, Abeer Zahir, Antonia Hassoulas, Eliana Panayiotou

**Affiliations:** 1 Cardiff University School of Medicine; 2 Swansea Bay University Health Board

## Abstract

**Aims:**

Since the COVID-19 outbreak was declared a global pandemic, public health messages have emphasised the importance of frequent handwashing in limiting the transmission of the virus. Whilst crucial in controlling transmission, such messaging may have an adverse effect on individuals with OCD. The primary aim of this study was to investigate any significant changes to handwashing behaviour, as well as other related hygiene behaviours, across all symptom dimensions of OCD. The frequency of engaging with pandemic-related media coverage was also considered across all symptom subtypes.

**Method:**

A cross-sectional study was conducted, with a total of 332 participants recruited. Participants who scored above the optimal cut-off score on the Obsessive-Compulsive Inventory Revised edition (OCI-R) were included in the analysis (n = 254). Scores on the six subscales of the OCI-R were correlated with responses to a COVID-19 Impact measure.

**Result:**

Factor analysis of the COVID-19 Impact measure revealed that items loaded on two components of the measure (handwashing and distress-avoidance). A significant correlation was revealed between the handwashing component and the OCI-R washing subscale (rs = 0.523, p = 0.0001), as well as between distress-avoidance and the OCI-R washing and ordering subscales (s = −0.227, p = 0.0001; rs = −0.159, p = 0.006). Content analysis revealed disruption to treatment delivery and worsening symptom severity in participants with contamination-related OCD.

**Conclusion:**

The pandemic has had a significant impact on individuals with contamination-related OCD symptoms, in relation to symptom severity and treatment disruption. Consideration should be given to targeted support tailored to patients with this subtype of OCD.

